# Analysis of cellulose synthase (CesA) promoter function in trees using Induced Somatic Sector Analysis (ISSA)

**DOI:** 10.1186/1753-6561-5-S7-O41

**Published:** 2011-09-13

**Authors:** Nicky Creux, Alexander Myburg, Gerd Bossinger, Antanas Spokevicius

**Affiliations:** 1 Department of Genetics, Forestry and Agricultural Biotechnology Institute (FABI), University of Pretoria, Pretoria, 0002, South Africa; 2 The University of Melbourne, Department of Forest and Ecosystem Science, Creswick, Victoria 3363, Australia

## 

Detailed knowledge of the tissue specificity of gene expression is of central importance not only for our understanding of developmental processes during wood formation, but also is a prerequisite for the deliberate manipulation of xylogenesis candidate genes. Today, much of our knowledge about specific gene expression is based on annual model plants in part because perennial tree systems are often seen as too cumbersome for detailed promoter studies. Here we address this issue in a novel way with the investigation of a number of well characterised cellulose synthase (CesA) genes in the stems of perennial tree species using the *in vivo* Induced Somatic Sector Analysis (ISSA) system. Three primary and four secondary cell wall associated CesA gene promoters from *Eucalyptus grandis* and *Arabidopsis thaliana* were previously isolated and cloned in front of the GUS reporter gene then, using *Agrobacterium*-mediated transformation, transformed into cambial initials in stems of eucalypt and poplar plants. A CAMV35S promoter::GUS vector was also used as a control. After a period of growth, stems were harvested and GUS-staining patterns were analysed and interpreted. Results from this work show that in eucalypts the staining patterns are consistent with observations in other species whereas in poplar conflicting results were observed. These findings and the general utility of ISSA for these studies are discussed.

